# VULNERABILITY PROFILES: CHALLENGES FOR METACOGNITIVE INTERVENTIONS WITH OLDER ADULT

**DOI:** 10.1093/geroni/igae098.2660

**Published:** 2024-12-31

**Authors:** Ann Pearman, Christopher Hertzog, MacKenzie Hughes, Emily Giannotto

**Affiliations:** MetroHealth Medical System, Cleveland, Ohio, United States; Georgia Institute of Technology, Atlanta, Georgia, United States; NORC at the University of Chicago, Chicago, Illinois, United States; Emory University, Atlanta, Georgia, United States

## Abstract

The metacognitive intervention approach has great promise for improving older adults’ everyday functioning. Results from our studies (Hertzog et al., 2021; Pearman et al., 2020; Pearman et al., 2023) and many others point to potential issues with intervention compliance and realization of intervention benefits in some older adults. Based on the first author’s clinical geropsychology work with older populations and observing our study participants, we argue that there are individual differences in who benefits from interventions and why they do so. This presentation will focus on our everyday memory training approach to evaluate variables that influence training benefit, including rates of attrition/drop-out, compliance with instructed task training, and improvements on outcome variables. Individual difference variables of interest include global cognition as measured by the Telephone Interview for Cognitive Status (TICS) and personality traits as measured by the AB5C with a particular focus on neuroticism and conscientiousness. The TICS was related to objective performance improvement on an associated learning task for both the intervention as well as the control group. Higher TICS scores were related to less improvement from T1 to T2 (r = -.39, p <.05). Personality variables correlated with post-test subjective memory but only in the intervention group, where people high in neuroticism scored lower on memory self-efficacy and higher on memory complaint [r = -.45 & r =.41 (respectively), p <.05]. These findings suggest that specific personal characteristics present barriers to adoption and maintenance of trained metacognitive strategies.

